# Development of the FAO/INFOODS/IZINCG Global Food Composition Database for Phytate

**DOI:** 10.1016/j.jfca.2019.01.023

**Published:** 2019-05

**Authors:** Sergio Dahdouh, Fernanda Grande, Sarah Nájera Espinosa, Anna Vincent, Rosalind Gibson, Karl Bailey, Janet King, Doris Rittenschober, U. Ruth Charrondière

**Affiliations:** a International Zinc Nutrition Consultative Group, Barcelona, Spain; b Nutrition and Food Systems Division, Food and Agriculture Organization, Rome, Italy; c University of Otago, Otago, New Zealand; d Children's Hospital Oakland Research Institute, Oakland, USA

**Keywords:** Ca, calcium, CRM, certified reference material, EP, edible portion, EFSA, European Food Safety Authority, FAO, Food and Agriculture Organization, FCT/FCDB, Food Composition Table/Food Composition Database, Fe, iron, GIFT, FAO/WHO Global Individual Food consumption data Tool, HPLC, high-performance liquid chromatography, INFOODS, International Network of Food Data Systems, IPs, inositol phosphates, IP3, inositol triphosphate, IP4, inositol tetraphosphate, IP5, inositol pentaphosphate, IP6, inositol hexaphosphate, IZiNCG, International Zinc Nutrition Consultative Group, PHY:FE, phytic acid forms : iron ratio, PHY:ZN, phytic acid forms : zinc ratio, PhyFoodComp, Global Food Composition Database for Phytate, PHYT-, phytic acid - unknown or variable method, PHYT:FE, phytic acid (global) : iron ratio, PHYT:ZN, phytic acid (global) : zinc ratio, PHYTAC, phytic acid - old tagname, PHYTC-, phytic acid - unknown colorimetry, PHYTCA, phytic acid - by K-PHYT kit, PHYTCPP, phytic acid - by anion exchange, PHYTCPPI, phytic acid - by indirect precipitation, PHYTCPPD, phytic acid - by direct precipitation, PP, phytate phosphorus, PP-, phytate phosphorus - by unknown colorimetry, PPD, phytate phosphorus - by direct precipitation, PPI, phytate phosphorus - by indirect precipitation, RNI/RDI, recommended nutrient intake/recommended dietary intake, XP, conversion factor for phytate phosphorus, Zn, zinc, Antinutrient, Food composition database, Inositol phosphates, Iron, Phytate, Phytic acid, Phytic acid:iron ratio, Phytic acid:zinc ratio, Zinc

## Abstract

Phytate is widely distributed in the plant kingdom, and its significance for human nutrition has been often described. Data on phytate is available in very few composition tables, for a limited number of foods and mainly for raw products. With the aim of publishing the first global repository of analytical data on phytate, data on moisture, phytate, zinc, iron and calcium were compiled. Other aspects, such as the analytical method used, biodiversity and processing, were considered, and phytate: mineral ratios were calculated when possible. From a comprehensive literature search, over 250 references were compiled, generating 3377 entries: 39% for raw and 61% for processed foods. Most of the entries were for cereals (35%), followed by legumes (27%) and vegetables (11%). The most common analytical methods used were indirect precipitation (26%) and anion exchange (25%), while separate determination of IPs is the most recommended. The database can be used as a tool for nutrition workers to include into food composition tables and to develop programmes related to mineral deficiencies. These data will be useful for designing diets with enhanced mineral bioavailability and for improving the estimates for nutrient requirements. The database is available at the INFOODS (www.fao.org/infoods/infoods/tables-and-databases/en) and IZiNCG webpages (www.izincg.org).

## Highlights

• The database aims contributing to the reduction of iron and zinc deficiencies across the world and to a revision of nutrient requirements of zinc and iron.
• The database aims raising awareness on food-based approaches for increasing the bioavailability of iron and zinc in foods.
• Phytate’s analysis should be centered on separate inositol phosphate forms rather than only total phytate, as it often overestimates the phytate content.
• Phytate contents are diminished by processing methods, such as fermentation, cooking or soaking.

## 1 Introduction

There are approximately 2 billion people in the world who suffer from micronutrient deficiencies (Global Nutrition Report, 2016). An estimated 17.3% of the world’s population is at risk of inadequate zinc intake (Wessells and Brown, 2012) while almost 30% are anaemic, many due to iron deficiency (WHO, 2013). Thus, both zinc and iron deficiencies constitute a significant public health problem.

Phytate is the storage form of phosphorus in plants and is found in high concentrations in seeds, cereals and pulses to allow the future germ to sprout adequately using its own nutrients, including the stored phosphorus. Since phytate cannot be degraded by endogenous enzymes of humans, and owing to its high mineral binding capacity due to the double charged phosphate groups, phytate binds cations and impedes their absorption (Gupta et al., 2015). Thus, phytate is often classified as an antinutrient. It is important to note that phytate has also been considered as a natural antioxidant by some authors, mainly by the virtue of forming a unique iron chelate that suppresses iron-catalysed oxidative reactions (Graf and Eaton, 1990; Empson et al., 1991). Phytate is one of the important compounds to be considered when determining the bioavailability of zinc and iron from different diets and the required dietary intake levels. For example, the recommended nutrient intakes (RNIs) for zinc and iron are about 3-times higher for diets with a low bioavailability compared to those with a high bioavailability for all age groups (FAO/WHO, 2004). Most of the phytate data available at the time of the Food and Agriculture Organization (FAO) and the World Health Organization (WHO) expert consultation on vitamin and mineral requirements (1998) were on total phytate content in the same raw foods (FAO, 2001).

The high RNIs for iron and zinc make it very difficult for individuals consuming plant-based diets to achieve their RNIs through foods alone. The low bioavailability of the minerals bound to the phytic acid can lead to deficiencies in human populations where wheat, rice and maize are consumed as staple foods (Bohn et al., 2008; Gibson and Ferguson, 1998; Gibson and Hotz, 2001).

Phytate refers to phytic acid — myoinositol hexaphosphate (IP6) — made up of an inositol ring with six phosphate ester groups, and its associated salts: magnesium, calcium, or potassium phytate (Gibson et al., 2006, 2010). However, there are five other inositol phosphates (IPs), each of which is named according to the number of phosphate groups attached to the inositol ring (from IP1 to IP6). The cation-binding capacity is a function of the number of phosphate groups on the inositol ring and their position. Available evidence indicates that phytate in pulses, cereals and other products can be degraded by simple processing methods, such as soaking, germination, and fermentation through converting IP6 to lower IPs, which interfere less with the bioavailability of zinc and iron (Schlemmer et al., 2009).

There have been very few cases where phytate data have been included in food composition tables and databases (FCT/FCDBs) and, in most such cases, the values included only represent the content for raw products, with no details of analytical methods used to generate the phytate values.

In 2016, FAO and the International Network of Food Data Systems (INFOODS), decided to compile phytate data from the literature for raw and processed foods. These data are intended to assist in re-evaluating certain assumptions concerning phytate and improve the basis for zinc and iron RNIs. The International Zinc Nutrition Consultative Group (IZiNCG) joined this process at a later stage and contributed expertise and funding received through the Bill and Melinda Gates Foundation project “*Development and assessment of intervention strategies to prevent zinc deficiency*” (Agreement No.: OPP1150161).

The objective of the FAO/INFOODS/IZiNCG Global Food Composition Database for Phytate (PhyFoodComp) is to report phytate contents together with those of selected minerals (iron, zinc and calcium), water, and phytate:mineral molar ratios, according to international quality standards. PhyFoodComp also aims to demonstrate the phytate decrease due to processing and the differences in phytate values due to different analytical methods. The database will thus provide a basis for recommending the most appropriate analytical methods for phytate determination and for establishing nutrient retention factors for different processing methods. This database has also the aim of helping designing and implementing better nutrition projects, programmes, interventions and policies aimed at reducing mineral deficiencies.

The database, including the complete list of references used for compilation, and the User Guide are freely available at the INFOODS-homepage (www.fao.org/infoods/infoods/tables-and-databases/en) and at the IZiNCG webpage (www.izincg.org).

## 2 Material and methods

### 2.1 Literature search and data collection

In 2016, FAO/INFOODS carried out a comprehensive literature search on the phytate content of different foods. Data sources included scientific papers, theses, university reports and FCT/FCDB. The papers were mainly obtained from an exhaustive Scopus search using the following keywords: phytate, phytic, inositol phosphate or ip6 (in title); and barley, bean, yam, vegetable, palm amaranth, seed, banana, baobab, leaf, leaves, beer, nut, flour, tuber, cassava, spice, fruit, cocoyam, kernel, fonio, lentil, pasta, maize, corn, millet, pea, potato, rice, sorghum, soy, taro, wheat, triticale, grain, cereal, bread, biscuit, food, vegetable, beverage, rapeseed, injera, gruel, porridge, bran, cracker, plantain, oats, rye, cake, pastry, cocoa, cacao, lupin, legume, pulse, teff or gram (in content). The information and the abstracts of the 6020 articles found were examined to determine the presence of useful data. Analytical data from five FCT/FCDB were also obtained, namely FAO/INFOODS Analytical Food Composition Database (FAO, 2017a); FAO/INFOODS Food Composition Database for Biodiversity (FAO, 2017b); Food Composition Table for use in The Gambia, 2011; Indian Food Composition Tables (Longvah et al., 2017); National Food Composition Tables and The Planning of Satisfactory Diets in Kenya (Sehmi, 1993).

### 2.2 Inclusion/exclusion of data and quality of data

All data were evaluated for data quality and presentation according to a set of quality criteria (Table 1). Exclusion criteria varied from imprecise food and analytical methodology descriptions, expression in dry matter basis without provision of water values, missing data and lack of units and denominators. Subsequently 72% of the papers originally selected were rejected.

**Table 1:** Examples for the exclusion of scientific articles for the Global Food Composition Database for Phytate.

Data description/presentation:
• Missing units and/or denominators
• Unreported or unclear basis (dry or fresh)
• Inconsistency in data presentation (e.g., in the article data refer to fresh weight basis but the corresponding table presents data on dry matter basis)
• Data presentation in graphs/figures, without providing related values
• Misleading table/graph description
Missing data needed to transform data to ‘per 100 g edible portion on a fresh weight basis’:
• Missing water content per 100 g edible portion of fresh weight basis (EP), if data were expressed as percentage or g of dry matter

Selected data quality checks, which are included in the FAO/ INFOODS Guidelines for Checking Food Composition Data prior to the Publication of a User Table/Database – version 1.0 (FAO/INFOODS, 2012) were applied to screen the data for consistency and reliability. These checks were constantly applied, both during the initial data evaluation and during the compilation of the database, in order to detect implausible data.

### 2.3 Standardisation of data

The standardisation aims to make uniform the expressions of components and their units and denominators, which allows a comparative evaluation and analysis of the data at a later stage. All nutrient values in PhyFoodComp are expressed per 100 g edible portion on a fresh weight basis (EP); therefore data found in other expressions needed to be converted to 100 g EP.

Data were compiled according to international standards for food composition and compilation, as outlined by Greenfield and Southgate (2003) using the FAO/INFOODS Compilation Tool (FAO, 2009), a simple food composition database management system in Microsoft Excel (Charrondière and Burlingame, 2011). The minerals and water were assigned to their respective INFOODS food component identifier, also called tagname (Klensin et al., 1989).

Since different chemical methods used to report phytate use distinct principles and analytical methods, they generate significantly different phytate values (Carlsson et al., 2001; Gao et al., 2007; Kasim and Edwards, 1998; Park et al., 2006; Rounds and Nielsen, 1993). Therefore, new tagnames had to be created prior to data compilation (Table 2). Advances in analytical methods allow the separation and determination of different inositol phosphates (IPs). For these methods, and depending on the degree of phosphorylation of the inositol, the tagnames assigned in the PhyFoodComp ranged from IP3 to IP6 — from three to six phosphate groups — for the individual IPs. Various combinations (sums) of IPs were also designated by different tagnames. The previous tagname ‘*Phytic Acid*’ (PHYTAC), which was used for all the different available methods for analysing total phytate, is now considered obsolete. Hence, all data that were previously reported under PHYTAC, were reclassified using the new tagnames by reviewing the original source. When a clear assignment to a tagname was not possible due to imprecise description of the analytical method, the tagname indicating ‘*unknown*’ was selected, i.e. PHYT-.

**Table 2:** Tagnames, description and units created for the Global Food Composition Database for Phytate.

New tagname	Description	Unit
PHYTCPPI	Phytic acid, determined by indirect precipitation	mg
PHYTCPPD	Phytic acid, determined by direct precipitation	mg
PHYTCA	Phytic acid, determined by colorimetry after an alkaline phosphatase hydrolysis	mg
PHYTCPP	Phytic acid, determined by anion exchange	mg
PHYTC-	Phytic acid, determined by colorimetry (unknown)	mg
PPI	Phytate phosphorus, determined by indirect precipitation	mg
PPD	Phytate phosphorus, determined by direct precipitation	mg
PP-	Phytate phosphorus, determined by colorimetry (unknown)	mg
XP	Conversion factor for phytate phosphorus	–
IP3	Inositol triphosphate	mg
IP4	Inositol tetraphosphate	mg
IP5	Inositol pentaphosphate	mg
IP6	Inositol hexaphosphate	mg
IP5_A_IP6	Inositol penta + hexaphosphate	mg
IP4_A_IP5_A_IP6	Inositol tetra + penta + hexaphosphate	mg
IPSUM	Total inositol phosphates (SUM of all IPs)	mg
PHYT-	Phytic acid, unknown or variable method	mg
PHYTCPPI:FE	Phytic acid (by indirect precipitation) : Iron ratio	–
PHYTCPPI:ZN	Phytic acid (by indirect precipitation) : Zinc ratio	–
PHYTCPPD:FE	Phytic acid (by direct precipitation) : Iron ratio	–
PHYTCPPD:ZN	Phytic acid (by direct precipitation) : Zinc ratio	–
PHYTCA:FE	Phytic acid (by K-PHYT kit) : Iron ratio	–
PHYTCA:ZN	Phytic acid (by K-PHYT kit) : Zinc ratio	–
PHYTCPP:FE	Phytic acid (by anion exchange) : Iron ratio	–
PHYTCPP:ZN	Phytic acid (by anion exchange) : Zinc ratio	–
PHYTC-:FE	Phytic acid (by unknown colorimetry) : Iron ratio	–
PHYTC-:ZN	Phytic acid (by unknown colorimetry) : Zinc ratio	–
PHYT-:FE	Phytic acid (by unknown method) : Iron ratio	–
PHYT-:ZN	Phytic acid (by unknown method) : Zinc ratio	–
PHY:FE	Phytic acid (by HPLC/HPAE) : Iron ratio	–
PHY:ZN	Phytic acid (by HPLC/HPAE) : Zinc ratio	–

To estimate the mineral binding effect of phytate on zinc and iron bioavailability the IZiNCG recommends the use of phytate:zinc molar ratios of the diet (Gibson et al., 2010), and Hurrell and Egli (2010) recommend the use of dietary phytate:iron molar ratios, respectively. Therefore, ratios for total phytate and IPs were calculated using the following formulas (Eqs. (1), (2), (3), (4)). Only IP4, IP5 and IP6 were used in the calculation of IPs:iron ratio (Eq. (4)), as these are the ones that can bind to the iron. For the IPs:zinc ratio equation, only IP5 and IP6 were considered (Eq. (3)), as no effect has been described with the lower IPs (Lönnerdal et al., 1989).

Eq. (1). PHYT:ZN formula

(1) $$\frac{\frac{Phytate\left(mg\right)}{660\;(MW)}}{\frac{Zn\left(mg\right)}{65.38(AtW)}}$$

Eq. (2). PHYT:FE formula

(2) $$\frac{\frac{Phytate\left(mg\right)}{660\;(MW)}}{\frac{Fe\left(mg\right)}{55.845(AtW)}}$$

Eq. (3). PHY:ZN formula

(3) $$\frac{\frac{IP6\left(mg\right)}{660\left(MW\right)}\;+\;\frac{IP5\left(mg\right)}{580\left(MW\right)}}{\frac{Zn\left(mg\right)}{65.38\left(AtW\right)}}\;$$

Eq. (4). PHY:FE formula

(4) $$\frac{\frac{IP6(mg)}{660(MW)}\;+\;\frac{IP5(mg)}{580(MW)}\;+\;\frac{IP4(mg)}{500\left(MW\right)}}{\frac{Fe\left(mg\right)}{55.845(AtW)}}$$

Where:

- Atw : Atomic weight

- MW : Molar weight

### 2.4 Compilation

All screened data were subsequently included in the FAO/INFOODS/IZINCG Global Food Composition Database for Phytate. Each of the foods was coded and categorised into one of the 19 food groups and their subgroups, adapted from the ‘*Food groups for simple indicators*’ classification system developed by FAO for the FAO/WHO Global Individual Food consumption data Tool (GIFT) platform (FAO, 2017a,b,c), which is based on the FoodEx2 food classification and description system (EFSA, 2015). In addition, every food was coded using FoodEx2, which is useful for harmonisation and linkage of food-related data across domains and for providing the possibility of semi-automated food matching.

## 3 Results

The FAO/INFOODS/IZiNCG Global Food Composition Database for Phytate (PhyFoodComp) is the first global repository containing only analytical data on the phytate content of foods. PhyFoodComp is an archival database according to Greenfield and Southgate (2003), which means that no nutrient or antinutrient values were calculated or estimated to complete the compositional profile of a food entry. The database often holds data of different edible parts of the same food; different processing stages (from raw to ultra-processed); different stages of maturity, growing and field conditions and storage; and also homemade and industrial complex recipes (composite foods). All data in PhyFoodComp are well documented, contain the food descriptions as in the original sources and additional information on processing in comment fields, so that users can utilise them without referring back to the original published sources.

Of the 6020 references found, data from 251 sources were compiled, generating 3377 individual food entries: 39% of the entries were for raw products and 61% for processed foods. A total of 15,412 component values are published in the database, of which the majority are minerals (33%), followed by phytic acid values, phytate phosphorus values and inositol phosphates (28%), ratios (20%), and water (19%).

Phytate data were not available for all the food groups and subgroups; therefore, some groups/subgroups remained empty, e.g., eggs. Zero values for phytate (e.g., some beverages, animal-source foods, etc.) were included in the database to emphasise its absence in these groups. In some cases, the assignment of a food to one specific food group was difficult, e.g., peanuts are botanically legumes but are considered as nuts in terms of their consumption and nutrient profile. This should be taken into consideration when searching for a food, as the assignment to a single food group might not be unequivocal. It is also recognised that the identification of the scientific names of species, subspecies and other lower species levels (especially for wild and underutilised foods), can often be difficult. English and scientific names are therefore presented as found in the original literature. Some sources use different names for the same food (e.g. maize or corn as English name for *Zea mays*), and they are listed as named in the original source.

**Fig. 1:**
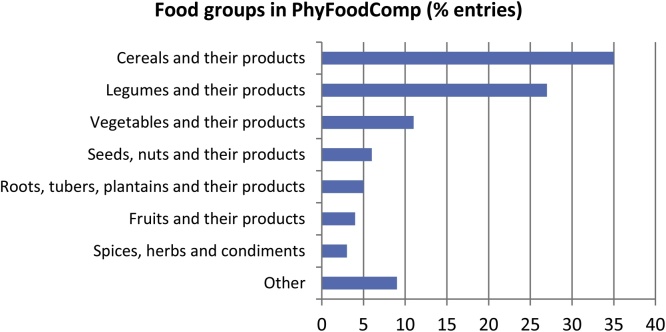
Main food groups in PhyFoodComp (% of entries).

From the 19 food groups considered for the PhyFoodComp, the majority of data correspond to cereals and their products (35%), followed by legumes and their products (27%), vegetables and their products (11%), seeds, nuts and their products (6%), roots, tubers, plantains and their products (5%), fruits and their products (4%), spices, herbs and condiments (3%) and the rest to other food groups (9%), such as complex recipes, beverages, food additives, etc. (Fig. 1).

The analytical methods for PHYTCPPI, PHYTCPPD, PHYTCA, PHYTCPP, PHYTC-, PHYT- are said to represent total IP6. These are usually based on indirectly measuring phosphorus from phytate and on the assumption that all the determined phosphate originated from the IP6, which is not always the case demonstrated by the different contents of IP3–IP6 when determined separately. The methods also assume that the phosphate has not been derived from other phosphorylated compounds that may exist. Hence, these procedures tend to overestimate the IP6 content of foods, especially plant-based foods and diets when food preparation or processing has resulted in varying degrees of phosphorylation and/or when other nucleotides are present (e.g., fermented foods). Consequently, these phytate values, intending to represent only IP6, are misleading in relation to the bioavailability of iron and zinc because their absorption is inhibited primarily by the IP6 and IP5.

**Fig. 2:**
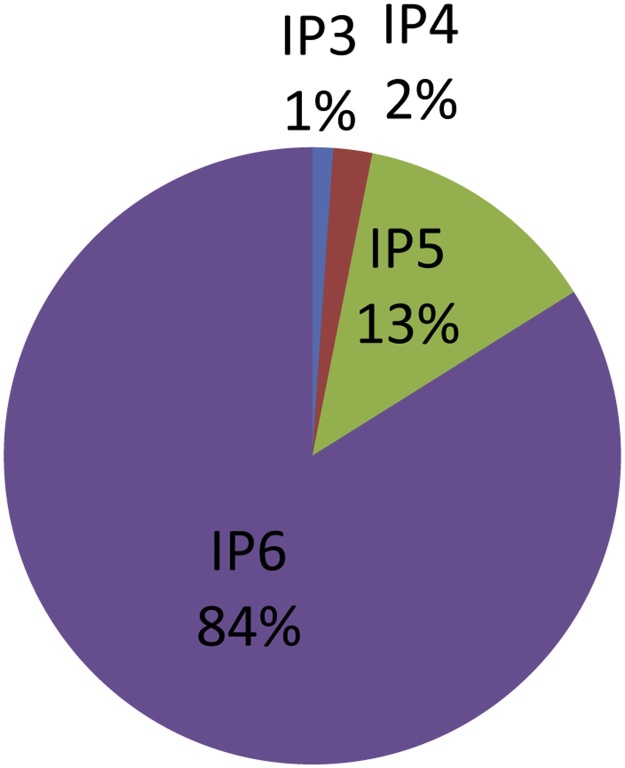
Amount of IPs in raw foods from PhyFoodComp.

Data of raw cereals, legumes and pulses in PhyFoodComp (Fig. 2) indicate that around 15% of the IPs amounts are present as lower IPs, mostly IP5 (≈13%), but also IP4 (≈2%) and IP3 (≈1%).

**Fig. 3:**
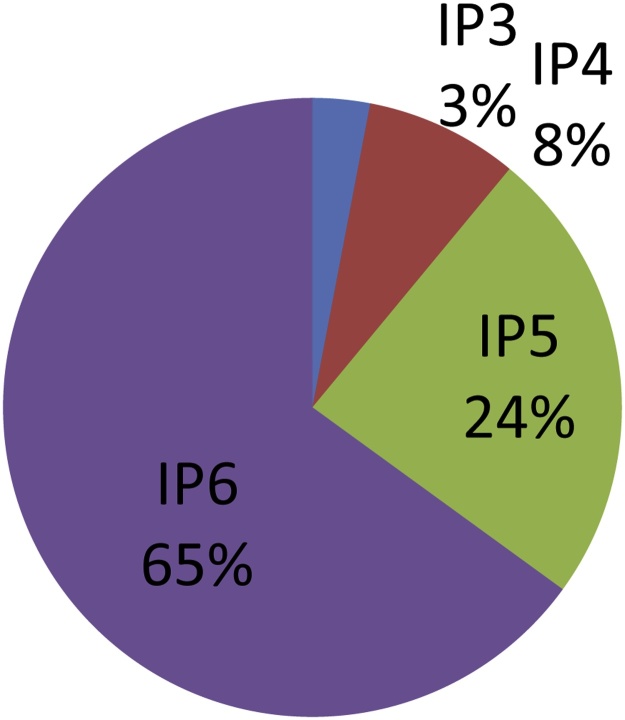
Amount of IPs in processed foods from PhyFoodComp.

In processed foods (i.e. fermented, boiled, soaked, etc.), significant amounts of IP6 are degraded to lower IPs, and therefore the relative amounts of IP5, IP4 and IP3 increase. In processed foods, a range between 3 and 84% of IPs (in cereals, legumes and pulses) are from lower IPs values compared to IP6. Often, the values are around 30–40% of lower IPs to IP6 (Fig. 3). Not only the relative percentage of lower IPs is increasing in processed products, but also the absolute amount of IP6 decreases with processing to 10–50% of the amounts in the corresponding raw food. In cereals and pulses, the IP6 amount in general is lower than any total phytate value.

When analysing the percentage of reduction of total phytate values when processing, it has been seen that some practices can largely reduce the total phytate content when compared to the raw commodity. Data show that, in some cases, processes such as fermenting or germinating can lead to a decrease of 40–65% and 50–80% of the total content in some pulses (e.g., cowpeas), respectively. Other processing methods, such as boiling or soaking, have also significant effects on phytate degradation, achieving reductions often between 20 and 40% in some cereals and pulses. Processes such as irradiation, extrusion, autoclaving, etc., can also be very effective when applied under certain conditions (i.e. long exposure, high temperature, etc.).

Coding the different entries using FoodEx2 significantly improved the quality of our datasets, as it made the data linkage and matching quicker, more robust and consistent and of higher quality. However, it is important to make clear that, as PhyFoodComp includes a lot of entries for the same foods and processings from different sources, the percentages and global averages presented cannot be taken as the rule. Differences between the samples (e.g., varieties, storage time), and the processing conditions (e.g., time, temperature, intensity) can lead to inconclusive or even confusing and nonsense results when analysed overall. Specific studies with the exact same samples and conditions should be taken or developed, in order to express more representative and reliable conclusions when needed. This is also the reason why it was not possible to estimate numerical differences between the different analytical methods (e.g., which total phytate method overestimates and by how much the phytate content, when compared to IP6).

When comparing precipitation methods, indirect precipitation appears to be more convenient and rapid than direct methods. However, when the phytate level is low, it is subject to a larger error (Reddy et al., 1989) when compared to the other total phytate detection methods. The method designated by the tagname PHYTCPP, developed by Harland and Oberleas (1986), includes an additional step in which the phytate extract is first purified and concentrated by anion-exchange chromatography prior to converting it to phosphate, providing more specificity and a lower error. The phytic acid (IP6) content termed more correctly ‘*phytic acid equivalent*” is then calculated on the basis that 1 g phytic acid phosphorus is equivalent to 3.55 g phytic acid (IP6), assuming that all phosphorus in the food is present as IP6, which is, as demonstrated above, often a wrong assumption. Another colorimetric method described is based on an alkaline phosphatase hydrolysis (PHYTCA) that hydrolyses phosphate from any compound that has a terminal phosphate group attached to it (*ab83369 Alkaline Phosphatase Assay Kit*). This last method works on the assumption that the only phosphate containing compounds in the sample being analysed are phytate compounds. This could be a big assumption and, if there are other phosphate-containing compounds in the sample, then this will overestimate the total amount of phytate.

More specific -and recommended- methods of measuring the various IPs often involve high-performance liquid chromatography (HPLC). Several HPLC methods are available, each of which uses anion exchange columns to purify and concentrate the phytate extract, followed by HPLC to separate and detect the individual IPs (Schlemmer et al., 2009). The problems in the determination of individual IPs are the lability of the lower IPs and the difficulty in obtaining certified reference material (CRM) for IP4 and below. Therefore, many laboratories do not use this recommended method. It would however be very useful if stable CRM became commercially available and then more laboratories could apply this method.

**Fig. 4:**
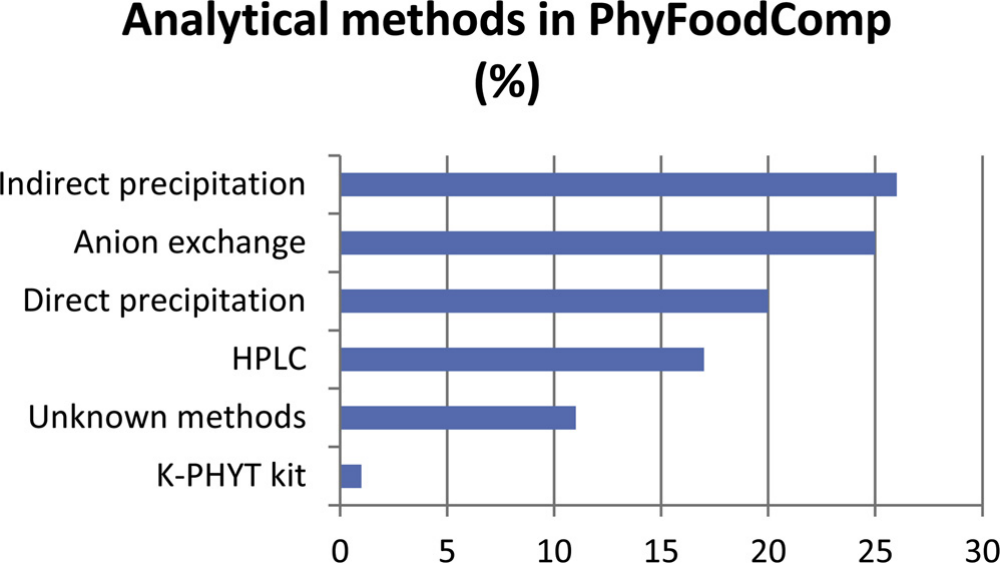
Analytical methods in PhyFoodComp (% of entries).

From the five analytical methods included in PhyFoodComp to report phytate, the majority of data correspond to indirect precipitation (26%), followed by anion exchange (25%), direct precipitation (20%), HPLC (17%), unknown methods (11%) and colorimetry after an alkaline phosphatase hydrolysis (1%), also known as K-PHYT kit (Fig. 4).

As mentioned above, IP6 represents the most abundant of the IPs (85%) found in mature unprocessed cereals, legumes, and oleaginous seeds. Consequently, for unprocessed plant-based foods, a non-specific method based on modifications of the ferric precipitation assay could be used. This will however overestimate the IP6 content by 10% because of the 15% lower IPs content. During certain processing practices (i.e. germination or fermentation) and storage, however, IP6 is usually dephosphorylated to lower IPs (i.e., IP5 to IP1), some of which no longer inhibit mineral absorption. The extent of the dephosphorylation depends on the duration and conditions (e.g., pH, temperature, humidity) of processing or storage, as well as the activity of the intrinsic phytase enzymes. In such cases, the non-specific total phytate methods will yield too high values that are overestimating their potential to inhibit mineral bioavailability. Therefore, instead, specific analytical methods (e.g., HPLC) that can separate and measure IP6 and IP5 from lower IPs should be used. It should however be emphasised that the IP6 will be degraded during storage to lower IPs and, thus, a determination of raw cereals, pulses and nuts using HPLC to determine IPs seems to be preferred also for these foods.

In PhyFoodComp, the calculation of 3103 ratios was carried out, of which 86% were compiled from entries where the phytate quantification was done by precipitation or unknown methods (PHYTCPPI, PHYTCPPD, PHYTCA, PHYTCPP, PHYTC-, PHYT-). These procedures provide phytate values, assumingly representing IP6, even though the phytate phosphorus (PP) is also released from IP4, IP5 and IP6. In the equations 2 and 3, the molar weight of IP6 (660 g/mol) is used in line with this assumption. This fact results in an overestimation of the phytate content of plant-based foods, especially if processed, as well as of the phytate:mineral ratios.

For the entries in which the specific amount of each IP was defined (IP4, IP5, IP6, IP5_A_IP6, IP4_A_IP5_A_IP6, IPSUM), the specific molecular weight corresponding to each IP (660, 580 and 500 for IP6, IP5 and IP4, respectively) was considered for the calculation of the phytate:mineral ratios, thus providing a more reliable evaluation of the binding effects on cereals, pulses and nuts.

The phytate to mineral molar ratios are used to predict the inhibitory effect of the antinutrient on the mineral bioavailability (Gibson et al., 1991). It is assumed that, for foods, the bioavailability of iron is affected by a ratio above 1, or even above 0.4 for a significant effect on absorption (Hurrell and Egli, 2010) because the inhibitory effect is observed at very low phytate concentrations (i.e., 2–10 mg in food/meal/diet) (Hallberg et al., 1989). In contrast, IZiNCG tentatively suggests that phytate:zinc molar ratios characterising unfermented, cereal-based diets (i.e., > 18) were likely to adversely affect zinc bioavailability (Brown et al., 2004). In the WHO/FAO semi-quantitative algorithm, diets were classified mainly on the basis of their source of protein and their phytate:zinc molar ratios, with ratios > 15 likely to compromise zinc bioavailability (WHO/FAO, 2004).

## 4 Discussion

The PhyFoodComp represents a comprehensive and expandable data repository of publicly available high-quality data with several uses (Table 3), where the variability of nutrient and antinutrient values, due to factors such as processing and biodiversity, is well portrayed.

**Table 3:** Uses of the Global Food Composition Database for Phytate.

For food composition:
• To allow compilers to include relevant phytate and mineral values into their FCTs/FCDBs
• To determine the differences in phytate values when using different analytical methods
For nutrition programs and policies:
• To give a new basis to revise assumptions on bioavailability and to revise the RDIs
• To provide the necessary data for developing apparent nutrient retention factors for different food groups and cooking/processing methods
• To enable governments and nutritionists to revise their advice on processing of foods, in order to increase the bioavailability of iron and zinc
• To provide the basis for advice regarding improvements in infant and young child feeding, diet formulations or product development
• To build an evidence-base for providing advice on processing methods to lower the phytate content and/or its mineral-binding capacity
• To raise awareness of food-based methods that increase the bioavailability of iron and zinc

Although several foods containing high phytate values were covered in PhyFoodComp, phytate data for other food groups and subgroups might become available in the near future. It is expected that more analytical data, e.g., centred on each separate IP (IP3–IP6) rather than only total phytate or with coverage of all relevant processing methods, will become available.

The next step would be the establishment of retention factors for phytate, based on various food processing practices that could be applied to mixed dishes and specific food and food subgroups, in an effort to improve the accuracy of the phytate values for prepared foods and diets. This will also provide much needed information on the most suitable processing methods per food category to reduce the phytate content and thus have the potential to increase the bioavailability of iron and zinc in plant-based foods and diets. Increasing the intake of bioavailable micronutrients through food-based approaches remains a highly sustainable means of ensuring the long-term prevention and treatment of micronutrient deficiencies. Such phytate-reducing processing strategies for raw, cooked and/or processed foods should be integrated into national food, agriculture, nutrition and health programmes/policies to enhance their effectiveness and sustainability (Gibson and Ferguson, 1998).

Studies suggest that IP6 and IP5 bind zinc and iron sufficiently strongly to inhibit both zinc and iron absorption (Gibson et al., 2010, 1991). Additional research is necessary to determine the specific affinity of iron to IP4 or IP3. Such affinity factors could then be used in the molar ratio equations, reflecting more closely the effect of the individual IPs on the bioavailability of iron, and possibly zinc. Studies on the relative affinity of total phytate or the different IPs might also be necessary, changing the relative amounts of each mineral to establish the relative affinity of phytate to different minerals. For example, what is the amount of iron bound, if huge amounts of calcium are present in the gut? It might be necessary to develop a phytate-equivalent for zinc, iron and calcium taking the absolute and relative affinity of the IPs to the different minerals into account.

On the other hand, It should be pointed out that the availability of Zn and Fe from food and diets does not just depend on the molar ratios of phytic acid:Zn or phytic acid:Fe, but can be affected by some other food compounds as well; e.g., ascorbic acid can enhance iron availability, while other food components, such as oxalic acid, may reduce it (Armah et al., 2013)-. Thus, it is highly desirable that further compounds affecting the availability of zinc and iron, such as polyphenols, ascorbic acid, oxalic acid, and the protein content, should be included in the database in the future to get a more reliable view on the availability of zinc and iron from food and diets. Determining the phytic acid:Zn and the phytic acid:Fe ratios of foods or meals alone might give limited information on the iron and zinc bioavailability in individuals or population groups.

## 5 Conclusion

The FAO/INFOODS/IZiNCG Global Food Composition Database for Phytate represents a comprehensive and expandable data repository of publicly available high-quality data, where the variability of nutrient and antinutrient values due to factors such as processing and biodiversity is well portrayed. The database, including the complete list of references used for compilation, and the User Guide are freely available at the INFOODS-homepage (www.fao.org/infoods/infoods/tables-and-databases/en) and at the IZiNCG webpage (www.izincg.org). It will hopefully be used to stimulate researchers and laboratories to determine IPs instead of total phytate; to revise RDIs of zinc and iron; to establish nutrient retention factors for different processing methods and finally will assist in developing improved recipes to prevent zinc and iron deficiencies worldwide.

It is expected that the database will contribute to the reduction of iron and zinc deficiencies across the world and will raise awareness of food-based approaches for increasing the bioavailability of these essential minerals in foods.
